# Fenofibrate attenuates doxorubicin-induced cardiotoxicity in patients with breast cancer: a randomized controlled trial

**DOI:** 10.1007/s00210-025-04326-1

**Published:** 2025-06-03

**Authors:** Hagar K. Dewidar, Amr A. Ghannam, Tarek M. Mostafa

**Affiliations:** 1 Clinical Pharmacy Department, Faculty of Pharmacy, Tanta University, Tanta, Egypt; 2 Clinical Oncology Department, Faculty of Medicine, Tanta University, Tanta, Egypt

**Keywords:** Doxorubicin, Fenofibrate, Cardiotoxicity, LVEF, NT-proBNP, MPO

## Abstract

Doxorubicin-induced cardiotoxicity (DIC) is a serious condition that limits its use. Thus, this study aimed at evaluating the efficacy and safety of fenofibrate in attenuating DIC in patients with breast cancer. In this randomized controlled parallel study, 44 patients with stage II and/or stage III breast cancer were randomly allocated into two groups: group 1 (control group; *n* = 22) which received doxorubicin/cyclophosphamide (AC regimen) for four cycles (cycle is every 3 weeks) and group 2 (fenofibrate group; *n* = 22) which received AC regimen for four cycles (cycle is every 3 weeks) plus 160 mg of oral fenofibrate 24 h prior to the first cycle of chemotherapy and then once daily until the end of the four chemotherapy cycles. At baseline and after the fourth chemotherapy cycle, all participants were submitted to echocardiography (echo) to evaluate left ventricular ejection fraction (LVEF) and blood sample collection to assess the serum levels of N-terminal pro-B-type natriuretic peptide (NT-proBNP), myeloperoxidase (MPO), alanine aminotransferase (ALT), and aspartate aminotransferase (AST). Data was analyzed using paired and unpaired* t*-tests, chi-square test, and Fisher exact test. Compared to baseline, the control group (AC) produced a significant increase in the serum levels of both NT-proBNP (*P* = 0.004) and MPO (*P* < 0.001). At the end of the study and as compared to the control group, the fenofibrate group showed significantly higher LVEF (*P* = 0.048) and a lower incidence of cardiotoxicity (*P* = 0.036). Additionally, the fenofibrate group showed a significant decline in the serum levels of NT-proBNP (*P* < 0.001) and MPO (*P* < 0.001). Moreover, fenofibrate was safe and well-tolerated and did not provoke a significant elevation in liver enzymes (*P* > 0.05). Fenofibrate could represent a promising prophylactic therapy against doxorubicin-induced cardiotoxicity. Trial registration: ClinicalTrials.gov ID: NCT06155331. Trial registration date 1-12-2023.

## Introduction

In 2022, breast cancer accounted for 11.6% of all cancer cases worldwide. With 666,000 deaths (6.9% of all cancer deaths), it ranks as the fourth most common cause of cancer mortality globally (Bray et al. 2024). Most triple negative (human epidermal growth factor receptor 2 (HER-2), progesterone receptor (PR), and estrogen receptor (ER) negative) breast cancers, as well as high-risk luminal HER-2 negative tumors, are treated with chemotherapy (Senkus et al. 2015). Doxorubicin (DOX) and/or taxanes represent the most often utilized chemotherapy regimens for the management of breast cancer (Early Breast Cancer Trialists’ Collaborative Group (EBCTCG 2012). Doxorubicin (DOX) is among the most effective chemotherapeutic agents used against breast cancer (Renu et al. 2018); however, its cardiotoxicity limits its usage (Swain et al. 2003).

Doxorubicin-related dose-dependent cardiotoxicity leads to myocardial structural damage and congestive heart failure (CHF). Even though the exact mechanisms through which DOX causes myocardial damage are still unclear, it is generally acknowledged that DOX causes cardiac damage through a number of mechanisms, including the activation of nuclear factor-kappa B (NF-ĸB), induction of pro-inflammatory cytokines, release of free radicals, encouragement of apoptotic cell death, and suppression of the mobilization and function of endothelial progenitor cells (EPCs) (Cardinale et al. 2020).

Transthoracic echocardiogram (echo), which is utilized for periodic evaluation of cardiac function and identification of any changes in left ventricular ejection fraction (LVEF), is the most often used diagnostic technique for detecting cardiotoxicity in oncological clinical practice (Al-hussaniy et al. 2023). For early prediction of cardiotoxicity in cancer patients, blood levels of N-terminal pro-B-type natriuretic peptide (NT-proBNP) and myeloperoxidase (MPO) can be measured (Ky et al. 2014; Lu et al. 2019). The cardiac ventricles produce Nt-proBNP in reaction to elevated wall strain (Song et al. 2005). It is considered an appropriate serum biomarker for the early identification of anthracycline-induced cardiotoxicity (Kittiwarawut et al. 2013). By encouraging the oxidation of sarcomeric protein and facilitating cardiac inflammation and apoptosis, MPO, a marker of inflammation, was reported to be elevated in patients with heart failure and cardiac dysfunction (Reichlin et al. 2010; Nettersheim et al. 2023). These aforementioned mechanisms led to the development of several treatments for anthracycline-induced cardiotoxicity, such as the implication of adrenergic receptor agonists and iron-chelating antioxidants; however, the side effects and the lack of satisfactory response of these treatments limit their use (Santos et al. 2018).

Endothelial dysfunction and decreased cardiac function are two characteristics of heart failure patients with poor circulation (Michowitz et al. 2007). The primary participants in endogenous healing processes that combat endothelial dysfunction are EPCs (Maltais et al. 2011). Peroxisome proliferator-activated receptor type alpha (PPARα) deficit may result in impaired functional capacity of the heart (Oka et al. 2015).

Fenofibrate, PPARα activator, has been shown to have a variety of pleiotropic actions on the heart that provide direct myocardial protection in addition to its lipid-lowering effects (Balakumar et al. 2011; Cheng et al. 2016). By endothelial nitric oxide synthase (eNOS) activation, short-term treatment with fenofibrate enhanced vascular endothelial function in middle-aged and older, healthy, normal lipidemic adults (Walker et al. 2012). In addition, fenofibrate’s anti-inflammatory properties protected against vascular dysfunction by enhancing the activity and mobilization of EPCs (Wang et al. 2014; Deng et al. 2017). Fenofibrate was also reported to exert beneficial effects in patients with systolic heart failure and can also attenuate cardiomyocyte hypertrophy by inhibition of NF-ĸB (Yin et al. 2013; Jen et al. 2016).

Therefore, we run this study to assess the efficacy and safety of fenofibrate in attenuating DOX-related cardiac toxicity in patients with breast cancer.

## Patients and methods

### Study design and patients’ selection

This study was a randomized controlled parallel study which was conducted on 44 patients with stage II and/or stage III breast cancer. Patients with breast cancer were recruited from the Clinical Oncology Department, Tanta University Hospital, Tanta, Egypt, between October 2023 and January 2025. The patients were simply randomized through the sealed envelope method into two groups: group 1 (the control group; *n* = 22) which received doxorubicin and cyclophosphamide “AC regimen” for four cycles (cycle every 3 weeks) and group 2 (fenofibrate group; *n* = 22) which received “AC regimen” for four cycles (cycle every three weeks) plus 160 mg of oral fenofibrate (Lipanthyl Supra®, MiniPharm Pharmaceuticals, Egypt) 24 h prior to the first cycle of chemotherapy and then once daily till the end of the four chemotherapy cycles. Doxorubicin dose was 60 mg/m^2^ which was diluted with 250 ml normal saline and was administered intravenously over 30 min, followed by cyclophosphamide 600 mg/m^2^ which was diluted with 500 ml normal saline and was administered intravenously over 60 min. Fenofibrate dose was the lowest safest dose used in the treatment of hypercholesterolemia and hypertriglyceridemia.

### Ethical approval

The study was conducted in accordance with the ethical standards declaration of Helsinki in 1964. The study was approved by the Research Ethics Committee of Tanta University (Approval Code: 36264MS391/1/11/23) and was registered as a clinical trial on ClinicalTrials.gov with ID (NCT06155331). All participants gave their written informed consent.

### Inclusion criteria

Female patients with biopsy-confirmed diagnosis of stage II and/or stage III breast cancer, age > 18 years old and < 65 years old, patients with performance status < 2 according to eastern Cooperative Oncology Group (ECOG) score, patients with adequate baseline hematologic values (absolute neutrophilic count ≥ 1.5 × 10^9^/L, platelet count ≥ 100 × 10^9^/L and hemoglobin level ≥ 10 g/dl), patients with adequate liver function (serum bilirubin < 1.2 mg), patients with adequate renal function (serum creatinine < 1.5 mg/dl), and patients with preserved left ventricular (LV) systolic function with LVEF ≥ 50%.

### Exclusion criteria

Patients with prior exposure to anthracyclines in the last 6 months, patients with evidence of metastasis at the initial assessment, concomitant use of antioxidant vitamins (vitamin A, C, E), patients with clinical evidence for severe cardiac illness (angina pectoris, uncontrolled hypertension, arrhythmias, and left ventricular ejection fraction < 50%), patients with inflammatory diseases (ulcerative colitis, rheumatoid arthritis), patients with conditions associated with oxidative stress (smoking, tuberculosis, comorbid obesity), patients who are candidates for monoclonal antibodies such as trastuzumab and other targeted therapy (HER2 positive patients), patients with active liver disease (cirrhosis, fatty liver, hepatitis C, etc.), patients with myopathy, patients with renal impairment including those with end-stage renal disease and those receiving dialysis, pregnant and breastfeeding women, patients with known allergy to fenofibrate, and patients on statin, ACEI, non-dihydropyridine CCBs, beta-blockers, liver microsomal enzyme inducers, liver microsomal enzyme inhibitors, and drugs with high plasma protein binding capacity were also excluded.

### Methods

#### Demography and physical examination

At baseline, all participants were submitted to demography, physical examination, family and medical history review, weight and height measurements, and calculation of both body mass index (BMI) and body surface area (BSA).

#### Clinical assessment (echocardiography and the incidence of cardiotoxicity)

Every patient was submitted to transthoracic echocardiogram (echo) 24 h before starting the medications (baseline) and after the end of the fourth chemotherapy cycles (after intervention) using (PHILIPS® Affiniti 70 ECHO machine) in order to measure left ventricular ejection fraction (LVEF). Cardiotoxicity is defined as the presence of heart failure symptoms with a reduction in ejection fraction by ≥ 5% from baseline or to less than 55% or the absence of heart failure symptoms with a reduction in ejection fraction by ≥ 10% from baseline or to less than 55% (Al-hussaniy et al. 2023).

#### Blood sample collection and assessment of biological markers

Five milliliters of venous blood were collected from each participant between 8:30 am and 10:30 am into plain test tubes at baseline (prior to initiation of chemotherapy) and after the fourth cycle of chemotherapy. Blood samples were allowed to clot and then centrifuged at 3000 rpm for 10 min. The separated sera were divided into two portions; the first portion was used for immediate determination of liver transaminases including alanine amino-transferase (ALT) and aspartate amino-transferase (AST) which were assayed spectrophotometrically. The second portion of the sera was kept frozen at − 80 °C until the analysis of the serum levels of N-terminal pro-B-type natriuretic peptide (NT-proBNP) and myeloperoxidase (MPO). The aforementioned biological markers were assessed using enzyme-linked immunosorbent assay (ELISA) kits which were based on the double-antibody sandwich ELISA technique and in accordance with the manufacturer’s instructions (SunRed Biological Technology Co., Ltd, Shanghai China with a catalogue numbers 201–12–1240 and 201–12-0881D for NT-proBNP and MPO, respectively).

#### Assessment of participants’ adherence and drug-related adverse effects

Patients were followed-up through weekly telephone calls and through planned meetings during chemotherapy cycles in order to assure their adherence and to report any drug (fibrate)-related adverse effects using the adverse drug reactions reporting form. The patients’ adherence was assessed by counting the number of returned pills. Patients are considered non-adherent if they consumed < 90% of the provided fenofibrate capsules throughout the study period. Non-adherent patients were excluded from the study.

#### Primary and secondary endpoints of the study

The primary endpoint was the change in left ventricular ejection fraction (LVEF).The secondary endpoint was the changes in serum levels of the measured biological markers (NT-proBNP and MPO).

#### Sample size calculation

Using SPSS program version 25 (SPSS Inc., Chicago, IL, USA, 2017) and with the assumption of a significance level of 0.05 (confidence interval of 95%) and statistical power of 80%, which in turn provided a large effect size of 0.930 to detect the difference in the outcome measured between the two groups using independent (unpaired) *t*-test, the total sample size was 40 patients in both arms (20 per group). Assuming that the attrition rate is 10%, the total sample size was 44 patients (22 patients per group).

#### Statistical analysis

Statistical analysis was done by the statistical software package SPSS version 25 (SPSS Inc., Chicago, IL, USA). Data were tested for normality using Shapiro–Wilk tests. Unpaired *t*-test was used to compare between the two study groups. Paired *t*-test was used to compare results within the same group before versus after treatment. Categorical data were computed by chi-square test. Fisher exact test was used to analyze the reported adverse effects. Correlation between variables was assessed by Pearson correlation. Numerical variables were expressed by mean ± standard deviation. Nominal variables were expressed by frequency and percentage. The significance level was set at *p* ≤ 0.05.

## Results

**Fig. 1:**
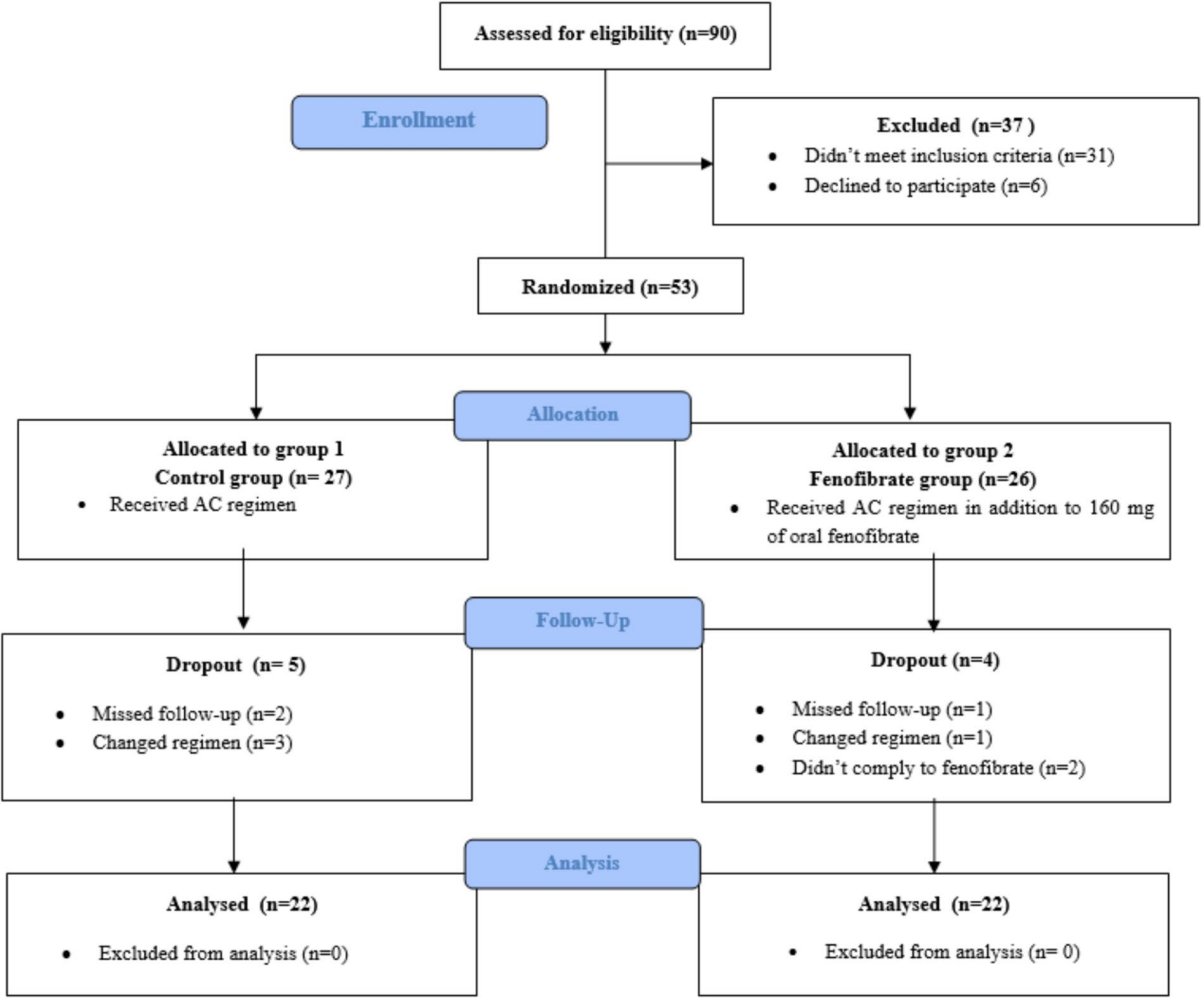
Study participant flowchart

Figure 1 illustrates the participant flowchart. Out of 90 patients with stage II and/or stage III breast cancer screened for eligibility, 37 patients were excluded (6 patients declined to participate and 31 patients did not meet the inclusion criteria). Therefore, the remaining 53 patients were randomized and assigned into the 2 study groups: group 1 (the control group; *n* = 27) and group 2 (fenofibrate group; *n* = 26). During the study period, five patients in the control group were dropped out secondary to loss of follow-up (*n* = 2) and change of chemotherapy regimen (*n* = 3) and four patients in the fenofibrate group were also dropped out as a result of loss of follow-up (*n* = 1), change of chemotherapy regimen (*n* = 1), and non-compliance with fenofibrate (*n* = 2). In this context, the final analysis involved 44 patients in both groups, with 22 patients in each group.

### Demographic, clinical, and laboratory data

The baseline demographic clinical and laboratory data of the two study groups were not significantly different, as demonstrated in Table 1. There was a non-significant difference regarding age, body surface area (BSA), body mass index (BMI), stage of breast cancer, side of breast cancer, family history of previous cancerous conditions, menopausal status, and previous use of contraceptives. In addition, the two groups were statistically similar regarding serum levels of liver enzymes including alanine amino-transferase (ALT) and aspartate amino-transferase (AST) both before (baseline) and after intervention as postulated in Table 1.

**Table 1:** Demographic, clinical, and laboratory data of the two study groups

Parameter		Control group (*n* = 22)	Fenofibrate group (*n* = 22)	*P*_2_-value
Age (year)		51 ± 9.55	50.41 ± 10.48	0.846
BSA (m^2^)		1.79 ± 0.22	1.78 ± 0.29	0.891
BMI (kg/m^2^)		33.87 ± 4.28	33.13 ± 5.39	0.616
Stage of breast cancer	Stage II	12 (54.5%)	11(50%)	0.763
	Stage III	10 (45.5%)	11 (50%)	
Side of breast cancer	Right	14 (63.6%)	13 (59.1%)	0.757
	Left	8 (36.4%)	9 (40.9%)	
Family history	Yes	5 (22.7%)	4 (18.2%)	0.709
	No	17 (77.3%)	18 (81.8%)	
Menopause	Pre-menopausal	11 (50%)	10 (45.5%)	0.763
	Post-menopausal	11 (50%)	12 (54.5%)	
Contraceptive	Yes	6 (27.3%)	4 (18.2%)	0.472
	No	16 (72.7%)	18 (81.8%)	
Diabetes	Yes	5 (22.7%)	6 (27.3%)	0.728
	No	17 (77.3%)	16 (72.7%)	
ALT (U/L)				
Baseline		21.82 ± 9.56	22.36 ± 8.35	0.841
After treatment		30.32 ± 10.71	29.41 ± 9.79	0.770
* P*_1_-value		< 0.001**	0.003*	––––
AST (U/L)				
Baseline		26.73 ± 7.98	26.14 ± 10.05	0.830
After treatment		27 ± 3210.11	33.82 ± 10.87	0.628
* P*_1_-value		0.010*	0.024*	––––

Data expressed as mean ± standard deviation, number, and percentage (%)

Control group: breast cancer patients treated with doxorubicin/cyclophosphamide (AC regimen). Fenofibrate group: breast cancer patients treated with doxorubicin/cyclophosphamide (AC regimen) plus fenofibrate

*BSA* body surface area, *BMI* body mass index, *ALT* alanine amino-transferase, *AST* aspartate amino-transferase, *kg* kilogram, *m*^*2*^ meter squared, *U/L* unit per liter

Significance level was set at *P* ≤ 0.05

*P*_1_-value: Probability of significance within the same group (before versus after treatment)

*P*_2_-value: Probability of significance between the two groups

*Statistically significant difference at *P* ≤ 0.05

### Clinical assessment

#### LVEF throughout the study period

The analysis of data at baseline using unpaired *t*-test revealed the absence of a significant difference in left ventricular ejection fraction (LVEF) between the two groups under study (*P* = 0.94). The analysis of data after intervention using paired *t*-test showed that both the control group and the fenofibrate group produced a significant decline in LVEF as compared to baseline data (*P*_1_ < 0.001 and *P*_1_ < 0.001, respectively). The comparison between the two groups after intervention using unpaired *t-*test showed that the fenofibrate treated group had a significantly higher LVEF as compared to the control group (*P* = 0.048) as postulated in Table 2.

**Table 2:** Change in ejection fraction (%) for the two study groups throughout the study period

Ejection fraction (%)	Control group (*n* = 22)		Fenofibrate group (*n* = 22)		*P*_2_-value
	Mean ± SD	Range	Mean ± SD	Range	
Baseline	64.86 ± 4.11	55–74	64.95 ± 3.86	59–74	0.94
After 3 months	59.50 ± 3.56	52–66	61.77 ± 3.83	55–73	0.048*
*P*_1_-value	< 0.001**		< 0.001**		––––

Control group: breast cancer patients treated with doxorubicin/cyclophosphamide (AC regimen). Fenofibrate group: breast cancer patients treated with doxorubicin/cyclophosphamide (AC regimen) plus fenofibrate.

Significance level was set at *P* ≤ 0.05

*P*_1_-value: Probability of significance within the same group (before versus after treatment)

*P*_2_-value: Probability of significance between the two groups

*Statistically significant difference at *P* ≤ 0.05

**Highly significant at *P* < 0.001

#### Incidence of cardiotoxicity

At the end of the treatment, no one of the patients (0%) in fenofibrate-treated group showed cardiotoxicity, while four patients (18.2%) in the control group showed doxorubicin-related cardiotoxicity since their ejection fractions were decreased by ≥ 10% from baseline values, resulting in a significant difference (*P* = 0.036) between the two study groups regarding the incidence of cardiotoxicity, as postulated in Table 3.

**Table 3:** Incidence of cardiotoxicity for the two study groups throughout the study

Incidence of cardiotoxicity	Control group (*n* = 22)	Fenofibrate group (*n* = 22)	*P*-value
Yes	4 (18.2%)	0 (0%)	0.036*
No	18 (81.8%)	22 (100%)	

Control group: breast cancer patients treated with doxorubicin/cyclophosphamide (AC regimen). Fenofibrate group: breast cancer patients treated with Doxorubicin/Cyclophosphamide (AC regimen) plus fenofibrate

Significance level was set at *P* ≤ 0.05

*P*-value: Probability of significance between the two groups

*Statistically significant difference at *P* ≤ 0.05

### Change in the serum levels of the biological markers for the two study groups

At baseline, the serum levels of NT-proBNP and MPO did not differ significantly between the two study groups (*P*_2_ > 0.05). After intervention and as compared to baseline data, the control group showed significant elevation of the serum levels of both NT-proBNP (*P*_1_ = 0.004) and MPO (*P*_1_ < 0.001). On the other hand, after intervention and as compared to the baseline values, the fenofibrate group showed significant reductions in the serum levels of both NT-proBNP (*P*_1_ = 0.011) and MPO (*P*_1_ = 0.031) as shown in Table 4. The comparison between the two study groups after the end of the study revealed that the fenofibrate group showed a significant decline in the serum levels of both NT-proBNP (*P*_2_ < 0.001) and MPO (*P*_2_ < 0.001) when compared to the control group as postulated in Table 4.

**Table 4:** Change in the levels of NT-proBNP and MPO for the two study groups

Parameters	Control group (*n* = 22)	Fenofibrate group (*n* = 22)	*P*_2_-value
	Mean ± S.D	Mean ± S.D	
NT-proBNP (pg/ml)			
Baseline	55.74 ± 9.77	55.35 ± 9.83	0.896
After treatment	64.85 ± 13.84	47.98 ± 9.77	< 0.001**
* P*_1_-value	0.004*	0.011*	––––
MPO (ng/ml)			
Baseline	36.24 ± 5.84	34.57 ± 6.19	0.362
After treatment	41.98 ± 6.60	29.95 ± 8.09	< 0.001**
* P*_1_- value	< 0.001**	0.031*	––––

Data expressed as mean ± standard deviation

Control group: breast cancer patients treated with doxorubicin/cyclophosphamide (AC regimen). Fenofibrate group: breast cancer patients treated with doxorubicin/cyclophosphamide (AC regimen) plus fenofibrate

*NT-proBNP* N-terminal pro-B-type natriuretic peptide, *MPO* myeloperoxidase, *pg/ml* pictogram per milliliter, *ng/ml* nanogram per milliliter

Significance level was set at *P* ≤ 0.05

*P*_1_-value: Probability of significance within the same group (before versus after treatment)

*P*_2_-value: Probability of significance between the two groups

*Statistically significant difference

*Significant *P* < 0.05

**Highly significant at *P* < 0.001

### Correlations between LVEF and serum biomarkers

After treatment, the control group exerted a significant negative relationship between LVEF and NT-proBNP (*r* = − 0.944; *P* < 0.001) and MPO (*r* = − 0.964; *P* < 0.001). On the other hand, after treatment, the fenofibrate group showed a significant positive association between LVEF and NT-proBNP (*r* = 0.948; *P* < 0.001) and MPO (*r* = 0.961; *P* < 0.001). Additionally, a significant positive association between NT-proBNP and MPO was observed in both the control group (*r* = 0.916; *P* < 0.001) and the fenofibrate group (*r* = 0.981; *P* < 0.001).

### Drug-related adverse effects

The analysis of data obtained with the current study revealed the absence of a significant difference between the control and fenofibrate groups regarding the reported adverse effects, as demonstrated in Table 5.

**Table 5:** The reported adverse effects for the two study groups

Adverse effects	Control group (*n* = 22)	Fenofibrate group (*n* = 22)	*P*-value
Nausea	20 (90.9%)	21 (95.5%)	0.549
Abdominal pain	7 (31.8%)	6 (27.3%)	0.746
Dizziness	2 (9.09%)	3 (13.6%)	0.641
Headache	4 (18.18%)	2 (9.1%)	0.386
Asthenia	21 (95.5%)	22 (100%)	0.32

Data expressed as number and percentage (%)

Control group: breast cancer patients treated with doxorubicin/cyclophosphamide (AC regimen). Fenofibrate group: breast cancer patients treated with doxorubicin/cyclophosphamide (AC regimen) plus fenofibrate

Significance level was set at *P* ≤ 0.05

## Discussion

Doxorubicin (DOX) represents an effective anticancer agent for multiple cancer types. However, its potential cardiotoxicity restrains its wide use in cancer management. In fact, doxorubicin-related cardiotoxicity is divided into three categories based on the time of onset. The first is acute cardiotoxicity, which occurs within 2 weeks after the initiation of chemotherapy or during treatment with chemotherapy and can be presented by electrocardiograph (ECG) changes, left ventricular dysfunction, and a decline in the left ventricular ejection fraction (LVEF). The second doxorubicin-related cardiotoxicity is early-onset chronic cardiotoxicity, which occurs within 1 year after stopping the treatment, usually manifesting as heart failure (HF) caused by dilated cardiomyopathy (DCM). The third doxorubicin-related cardiotoxicity is late-stage chronic cardiotoxicity, which develops years or even decades after the end of chemotherapy (Wenningmann et al. 2019; Raj et al. 2014; Zamorano et al. 2016). In this context, during the current study, doxorubicin provoked acute cardiotoxicity, which was presented by a significant decline in the left ventricular ejection fraction (LVEF) which in turn is considered an early indicator of doxorubicin-induced cardiotoxicity (Pardo Sanz and Zamorano 2020). Moreover, some previous studies demonstrated that acute doxorubicin cardiotoxicity is associated with frequent and significant decreases in LVEF (Dulf et al. 2023).

Since doxorubicin (DOX) is a commonly used antineoplastic that works against a variety of malignant disorders, including breast cancer, the purpose of this study was to assess the potential effectiveness and safety of fenofibrate in attenuating DOX-related cardiac toxicity in patients with breast cancer (Renu et al. 2018).

As there are no previous studies in the literature, according to the best of the authors’ knowledge, aimed at evaluating the cardioprotective effect of fenofibrate in patients receiving doxorubicin, we used the 160 mg daily dose of fenofibrate as an average and trial dose. Additionally, and despite it not being the same clinical setting, this selected dose of fenofibrate (160 mg daily) and the selected duration of use (12 weeks) were based on previous studies that evaluated the anti-inflammatory and antioxidant activity of 160 mg daily of fenofibrate in patients with metabolic syndrome (Rosenson et al. 2007; Rosenson 2009).

The most popular technique for cardiac monitoring during the implication of DOX is the serial evaluation of LVEF (Al-hussaniy et al. 2023). The presence of heart failure symptoms with an ejection-fraction reduction of ≥ 5% from baseline or to less than 55%, or the absence of symptoms with an ejection-fraction reduction of ≥ 10% from baseline or to less than 55%, is referred to as cardiotoxicity (Herrmann et al. 2022). According to these aforementioned criteria, at the end of treatment, the fenofibrate-treated group showed a significantly higher LVEF than the control group, and none of the patients in this group developed cardiotoxicity, whereas four patients (18.8%) in the control group showed doxorubicin-related cardiotoxicity. Our results regarding LVEF are consistent with Huang et al.’s, who revealed that fenofibrate treatment improved LV function by promoting the circulation of endothelial progenitor cells (EPC) and activating cardiac nitric oxide (NO) with a subsequent reduced inflammatory response, which in turn resulted in cardio-protection against DOX in mice (Huang et al. 2021). Our results also seem to be in consonance with a prior study that demonstrated that fenofibrate reduced the heart damage induced by the implication of daunorubicin (Jen et al. 2017).

Compared to echocardiography, natriuretic peptides (NPs) are thought to be more sensitive indicators for doxorubicin-induced cardiotoxicity “DIC” (Suzuki et al. 1998). Although LVEF did not significantly change after doxorubicin administration in a prior prospective trial, NT-pro-BNP level was considerably raised at several time points following the end of treatment (De Iuliis et al. 2016). Additionally, it was noted that the level of natriuretic peptides (NPs) tended to be raised within 24 h after anthracyclines exposure, which makes NPs level a good tool to identify acute cardiotoxicity (Romano et al. 2011; Lenihan et al. 2016). Moreover, the severity of heart failure was reported to be correlated with the level of NT-proBNP (Yu et al. 1996; Selvais et al. 1998). The data obtained with the current study proved that, after treatment, the control group showed a significant increase in NT-proBNP level when compared to its baseline value. Additionally, we observed the presence of a significant negative correlation between LVEF and NT-proBNP level. Our aforementioned results come in the same line with the findings reported by other authors who stated that serum levels of NT-proBNP were elevated in patients with varying degrees of heart failure and were linked to the drop in LVEF (Clerico et al. 1998). At the end of the current study, NT-proBNP level was significantly lower in the fenofibrate group than in the control group, a result that seems consistent with a previous research demonstrating that fenofibrate dramatically reduced NT-proBNP level when compared to the control in a mice model of DOX-induced cardiotoxicity (Huang et al. 2021). Our previously mentioned result can be explained on the basis of the anti-inflammatory properties of fenofibrate, which can inhibit cardiomyocyte hypertrophy through reducing NF-κB activation (Jen et al. 2016).

Patients with heart failure and cardiac dysfunction have higher levels of MPO, a marker of inflammation which is well-known to be linked to the severity of the disease (Ky et al. 2014). Because of its enzymatic activity, MPO is associated with the inflammatory response which in turn can result in tissue damage (Frangie and Daher 2022). It also contributes significantly to DOX-induced cardiotoxicity, in which the drug causes infiltration of cardiac neutrophils and production of MPO, which directly reduces cardiac contractility, cardiac inflammation, and cardiomyocyte death (Nettersheim et al. 2023). Additionally, MPO seems crucial for NO scavenging and NO synthase inhibition (Mukhopadhyay et al. 2009). Additionally, it was mentioned that cardiac dysfunction can be prevented by MPO genetic ablation or medication-based MPO suppression (Nettersheim et al. 2023). The findings from the current study showed that, after treatment, the control group showed a significant elevation in MPO level when compared to its baseline value. Additionally, we noticed the presence of a significant negative correlation between LVEF and MPO level. Our previously mentioned results come in matching with the findings reported by other researchers, who reported that serum level of MPO was significantly elevated after DOX administration and MPO level was linked to cardiotoxicity in patients with breast cancer who received DOX (Ky et al. 2014). Furthermore, after treatment, the MPO level in fenofibrate-treated patients was significantly lower than the control group, a result which comes in agreement with the findings of a prior clinical study that demonstrated the antioxidant role of fenofibrate and its capacity to improve vascular endothelial function through significant reduction of MPO levels in patients with type 2 diabetes mellitus (Nita et al. 2014). This could be explained on the basis that fenofibrate suppresses MPO secondary to its anti-inflammatory properties which is achieved through activation of endothelial nitric oxide synthase (eNOS) and through attenuating transactivation of NF-κB with subsequent increased bioavailability of nitric oxide (NO), which suppresses matrix metalloproteinase-2 “MMP-2,” matrix metalloproteinase-9 “MMP-9,” and tumor necrosis factor alpha “TNF-α” (Huang et al. 2021).

After treatment, the control group showed a significant negative association between MPO and NT-proBNP levels with LVEF, which is suggestive of the potential role of these biomarkers in chemotherapy-induced cardiotoxicity (Clerico et al. 1998; Rudolph et al. 2007). In contrast, fenofibrate-treated group showed a significant positive correlation between MPO and NT-proBNP levels with LVEF, whereas the implication of fenofibrate was associated with a non-significant reduction in LVEF as compared to its baseline, which was aligned with a significant reduction in both MPO and NT-proBNP levels.

The data obtained with the current clinical study revealed the absence of significant variation between the two study groups regarding the reported adverse effects. Furthermore and in contrast with some previous studies that reported that fenofibrate can provoke mild elevation in the liver transaminases (Roberts 1989; Balfour et al. 1990; Kobayashi et al. 2009) during our study, fenofibrate-treated group did not show significant elevation in both ALT and AST levels as compared to the control group, indicating that fenofibrate was safe and well-tolerated.

It is worth mentioning that, during the course of the study, patients in both the control group and the fenofibrate group did not need hospitalization secondary to doxorubicin-induced cardiotoxicity. Despite ejection fraction cutoff being a crucial factor in the clinical decision-making process, it does not determine the need for hospitalization on its own (Yancy et al. 2013). In addition, no further or long-term follow-up was done after the end of the study.

## Points of strength

The current study includes its design as a randomized controlled parallel study, the use of the same brand of fenofibrate throughout the study, and its priority as a first clinical study aimed at assessing the capacity of fenofibrate to attenuate doxorubicin-induced cardiac toxicity according to the best of the authors’ knowledge.

## Limitations

A relatively small sample size and being a single-center open-label study. In this context, future studies are still needed.

## Conclusion

This randomized controlled parallel study revealed the safety and tolerability of fenofibrate and its effectiveness in attenuating doxorubicin-related cardiac toxicity. The administration of fenofibrate by patients with breast cancer on doxorubicin-based chemotherapy was associated with a significant decline in the incidence of doxorubicin-induced cardiac toxicity. This favorable effect of fenofibrate on doxorubicin-induced cardiac toxicity is related to its anti-inflammatory effect since the implication of fenofibrate resulted in a significant decline in the serum level of myeloperoxidase as compared to the control group. Despite these promising results, future large-scale multi-center studies are still recommended.

## Data Availability

All source data for this work (or generated in this study) are available upon reasonable request.

## References

[CR1] Al-hussaniy HA, Alburghaif AH, Alkhafaje Z, et al (2023) Chemotherapy-induced cardiotoxicity: a new perspective on the role of Digoxin, ATG7 activators, Resveratrol, and herbal drugs. J Med Life 16:491–500. 10.25122/jml-2022-0322 10.25122/jml-2022-0322 PMC10251384 37305823

[CR2] Balakumar P, Rohilla A, Mahadevan N (2011) Pleiotropic actions of fenofibrate on the heart. Pharmacol Res 63:8–12. 10.1016/j.phrs.2010.11.002 21093591 10.1016/j.phrs.2010.11.002

[CR3] Balfour JA, McTavish D, Heel RC (1990) Fenofibrate Drugs 40:260–290. 10.2165/00003495-199040020-00007 2226216 10.2165/00003495-199040020-00007

[CR4] Bray F, Laversanne M, Sung H et al (2024) Global cancer statistics 2022: GLOBOCAN estimates of incidence and mortality worldwide for 36 cancers in 185 countries. CA Cancer J Clin 74:229–263. 10.3322/caac.21834 38572751 10.3322/caac.21834

[CR5] Cardinale D, Iacopo F, Cipolla CM (2020) Cardiotoxicity of anthracyclines. Front Cardiovasc Med 7. 10.3389/FCVM.2020.00026 10.3389/fcvm.2020.00026 PMC7093379 32258060

[CR6] Cheng H, Xi Y, Chi X et al (2016) Fenofibrate treatment of rats with experimental autoimmune myocarditis by alleviating Treg/Th17 disorder. Central Eur J Immunol 1:64–70. 10.5114/ceji.2016.58817 10.5114/ceji.2016.58817 PMC4829822 27095924

[CR7] Clerico A, Iervasi G, Del Chicca MG et al (1998) Circulating levels of cardiac natriuretic peptides (ANP and BNP) measured by highly sensitive and specific immunoradiometric assays in normal subjects and in patients with different degrees of heart failure. J Endocrinol Invest 21:170–179. 10.1007/BF03347297 9591213 10.1007/BF03347297

[CR8] De Iuliis F, Salerno G, Taglieri L et al (2016) Serum biomarkers evaluation to predict chemotherapy-induced cardiotoxicity in breast cancer patients. Tumor Biol 37:3379–3387. 10.1007/s13277-015-4183-7 10.1007/s13277-015-4183-7 26449821

[CR9] Deng Y, Han X, Yao Z et al (2017) PPARα agonist stimulated angiogenesis by improving endothelial precursor cell function via a NLRP3 inflammasome pathway. Cell Physiol Biochem 42:2255–2266. 10.1159/000479999 28817808 10.1159/000479999

[CR10] dos Santos DS, dos Goldenberg RC, S, Santos DS dos, Goldenberg RC dos S, (2018) Doxorubicin-induced cardiotoxicity: from mechanisms to development of efficient therapy. Cardiotoxicity. 10.5772/INTECHOPEN.79588

[CR11] Dulf PL, Mocan M, Coadă CA, Dulf DV, Moldovan R, Baldea I, Farcas A-D, Blendea D, Filip AG (2023) Doxorubicin-induced acute cardiotoxicity is associated with increased oxidative stress, autophagy, and inflammation in a murine model. Naunyn-Schmiedeberg’s Arch Pharmacol 396(6):1105–1115. 10.1007/s00210-023-02382-z 36645429 10.1007/s00210-023-02382-z PMC10185623

[CR12] Early Breast Cancer Trialists’ Collaborative Group (EBCTCG) (2012) Comparisons between different polychemotherapy regimens for early breast cancer: meta-analyses of long-term outcome among 100 000 women in 123 randomised trials. Lancet 379:432–444. 10.1016/S0140-6736(11)61625-5 22152853 10.1016/S0140-6736(11)61625-5 PMC3273723

[CR13] Frangie C, Daher J (2022) Role of myeloperoxidase in inflammation and atherosclerosis (Review). Biomed Rep 16:53. 10.3892/br.2022.1536 35620311 10.3892/br.2022.1536 PMC9112398

[CR14] Herrmann J, Lenihan D, Armenian S et al (2022) Defining cardiovascular toxicities of cancer therapies: an International Cardio-Oncology Society (IC-OS) consensus statement. Eur Heart J 43:280–299. 10.1093/eurheartj/ehab674 34904661 10.1093/eurheartj/ehab674 PMC8803367

[CR15] Huang W-P, Yin W-H, Chen J-S et al (2021) Fenofibrate attenuates doxorubicin-induced cardiac dysfunction in mice via activating the eNOS/EPC pathway. Sci Rep 11:1159. 10.1038/s41598-021-80984-4 33441969 10.1038/s41598-021-80984-4 PMC7806979

[CR16] Jen H-L, Liu P-L, Chen Y-H et al (2016) Peroxisome proliferator-activated receptor α reduces endothelin-1-caused cardiomyocyte hypertrophy by inhibiting nuclear factor-κ B and adiponectin. Mediators Inflamm 2016:1–11. 10.1155/2016/5609121 10.1155/2016/5609121 PMC5078655 27807394

[CR17] Jen H-L, Yin W-H, Chen J-W, Lin S-J (2017) Endothelin-1-induced cell hypertrophy in cardiomyocytes is improved by fenofibrate: possible roles of adiponectin. J Atheroscler Thromb 24:508–517. 10.5551/jat.36368 27629528 10.5551/jat.36368 PMC5429166

[CR18] Kittiwarawut A, Vorasettakarnkij Y, Tanasanvimon S et al (2013) Serum <scp>NT</scp> -pro <scp>BNP</scp> in the early detection of doxorubicin-induced cardiac dysfunction. Asia Pac J Clin Oncol 9:155–161. 10.1111/j.1743-7563.2012.01588.x 22897825 10.1111/j.1743-7563.2012.01588.x

[CR19] Kobayashi A, Suzuki Y, Kuno H et al (2009) Effects of fenofibrate on plasma and hepatic transaminase activities and hepatic transaminase gene expression in rats. J Toxicol Sci 34:377–387. 10.2131/JTS.34.377 19652460 10.2131/jts.34.377

[CR20] Ky B, Putt M, Sawaya H et al (2014) Early increases in multiple biomarkers predict subsequent cardiotoxicity in patients with breast cancer treated with doxorubicin, taxanes, and trastuzumab. J Am Coll Cardiol 63:809–816. 10.1016/j.jacc.2013.10.061 24291281 10.1016/j.jacc.2013.10.061 PMC4286181

[CR21] Lenihan DJ, Stevens PL, Massey M et al (2016) The utility of point-of-care biomarkers to detect cardiotoxicity during anthracycline chemotherapy: a feasibility study. J Card Fail 22:433–438. 10.1016/j.cardfail.2016.04.003 27079675 10.1016/j.cardfail.2016.04.003

[CR22] Lu X, Zhao Y, Chen C et al (2019) BNP as a marker for early prediction of anthracycline-induced cardiotoxicity in patients with breast cancer. Oncol Lett. 10.3892/ol.2019.10827 31612011 10.3892/ol.2019.10827 PMC6781730

[CR23] Maltais S, Perrault LP, Ly HQ (2011) The bone marrow–cardiac axis: role of endothelial progenitor cells in heart failure☆. Eur J Cardiothorac Surg 39:368–374. 10.1016/j.ejcts.2010.04.022 20663680 10.1016/j.ejcts.2010.04.022

[CR24] Michowitz Y, Goldstein E, Wexler D et al (2007) Circulating endothelial progenitor cells and clinical outcome in patients with congestive heart failure. Heart 93:1046–1050. 10.1136/hrt.2006.102657 17277352 10.1136/hrt.2006.102657 PMC1955007

[CR25] Mukhopadhyay P, Rajesh M, Bátkai S et al (2009) Role of superoxide, nitric oxide, and peroxynitrite in doxorubicin-induced cell death in vivo and in vitro. Am J Physiol-Heart Circ Physiol 296:H1466–H1483. 10.1152/ajpheart.00795.2008 19286953 10.1152/ajpheart.00795.2008 PMC2685360

[CR26] Nettersheim FS, Schlüter JD, Kreuzberg W et al (2023) Myeloperoxidase is a critical mediator of anthracycline-induced cardiomyopathy. Basic Res Cardiol 118:36. 10.1007/s00395-023-01006-0 37656254 10.1007/s00395-023-01006-0 PMC10474188

[CR27] Nita C, Bala C, Porojan M, Hancu N (2014) Fenofibrate improves endothelial function and plasma myeloperoxidase in patients with type 2 diabetes mellitus: an open-label interventional study. Diabetol Metab Syndr 6:30. 10.1186/1758-5996-6-30 24594096 10.1186/1758-5996-6-30 PMC3974011

[CR28] Oka S, Zhai P, Yamamoto T et al (2015) Peroxisome proliferator activated receptor-α association with silent information regulator 1 suppresses cardiac fatty acid metabolism in the failing heart. Circ Heart Fail 8:1123–1132. 10.1161/CIRCHEARTFAILURE.115.002216 26443578 10.1161/CIRCHEARTFAILURE.115.002216 PMC4651813

[CR29] Pardo Sanz A, Zamorano JL (2020) ‘Cardiotoxicity’: time to define new targets? Eur Heart J 41(18):1730–1732. 10.1093/eurheartj/ehaa013 32016397 10.1093/eurheartj/ehaa013

[CR30] Raj S, Franco VI, Lipshultz SE (2014) Anthracycline-induced cardiotoxicity: a review of pathophysiology, diagnosis, and treatment. Curr Treat Options Cardiovasc Med 16:315. 10.1007/s11936-014-0315-4 24748018 10.1007/s11936-014-0315-4

[CR31] Reichlin T, Socrates T, Egli P et al (2010) Use of myeloperoxidase for risk stratification in acute heart failure. Clin Chem 56:944–951. 10.1373/clinchem.2009.142257 20413430 10.1373/clinchem.2009.142257

[CR32] Renu K, Abilash VG, PB TP, Arunachalam S (2018) Molecular mechanism of doxorubicin-induced cardiomyopathy – An update. Eur J Pharmacol 818:241–253. 10.1016/J.EJPHAR.2017.10.043 10.1016/j.ejphar.2017.10.043 29074412

[CR33] Roberts WC (1989) Safety of fenofibrate–US and worldwide experience. Cardiology 76:169–179. 10.1159/000174488 2673510 10.1159/000174488

[CR34] Romano S, Fratini S, Ricevuto E et al (2011) Serial measurements of NT-proBNP are predictive of not-high-dose anthracycline cardiotoxicity in breast cancer patients. Br J Cancer 105:1663–1668. 10.1038/bjc.2011.439 22068815 10.1038/bjc.2011.439 PMC3242597

[CR35] Rosenson RS (2009) Effect of fenofibrate on adiponectin and inflammatory biomarkers in metabolic syndrome patients. Obesity 17:504–509. 10.1038/oby.2008.530 19023279 10.1038/oby.2008.530

[CR36] Rosenson RS, Wolff DA, Huskin AL et al (2007) Fenofibrate therapy ameliorates fasting and postprandial lipoproteinemia, oxidative stress, and the inflammatory response in subjects with hypertriglyceridemia and the metabolic syndrome. Diabetes Care 30:1945–1951. 10.2337/DC07-0015 17483155 10.2337/dc07-0015

[CR37] Rudolph V, Rudolph TK, Hennings JC et al (2007) Activation of polymorphonuclear neutrophils in patients with impaired left ventricular function. Free Radic Biol Med 43:1189–1196. 10.1016/j.freeradbiomed.2007.07.016 17854714 10.1016/j.freeradbiomed.2007.07.016

[CR38] Selvais, Donckier, Robert et al (1998) Cardiac natriuretic peptides for diagnosis and risk stratification in heart failure: influences of left ventricular dysfunction and coronary artery disease on cardiac hormonal activation. Eur J Clin Invest 28:636–642. 10.1046/j.1365-2362.1998.00338.x 9767358 10.1046/j.1365-2362.1998.00338.x

[CR39] Senkus E, Kyriakides S, Ohno S et al (2015) Primary breast cancer: ESMO Clinical Practice Guidelines for diagnosis, treatment and follow-up. Ann Oncol 26:v8–v30. 10.1093/annonc/mdv298 26314782 10.1093/annonc/mdv298

[CR40] Song BG, Jeon ES, Kim YH et al (2005) Correlation between levels of N-terminal pro-B-type natriuretic peptide and degrees of heart failure. Korean J Intern Med 20:26. 10.3904/kjim.2005.20.1.26 15906950 10.3904/kjim.2005.20.1.26 PMC3891409

[CR41] Suzuki T, Hayashi D, Yamazaki T et al (1998) Elevated B-type natriuretic peptide levels after anthracycline administration. Am Heart J 136:362–363. 10.1053/hj.1998.v136.89908 9704703 10.1053/hj.1998.v136.89908

[CR42] Swain SM, Whaley FS, Ewer MS (2003) Congestive heart failure in patients treated with doxorubicin: a retrospective analysis of three trials. Cancer 97:2869–2879. 10.1002/CNCR.11407 12767102 10.1002/cncr.11407

[CR43] Walker AE, Kaplon RE, Lucking SMS et al (2012) Fenofibrate improves vascular endothelial function by reducing oxidative stress while increasing endothelial nitric oxide synthase in healthy normolipidemic older adults. Hypertension 60:1517–1523. 10.1161/HYPERTENSIONAHA.112.203661 23108655 10.1161/HYPERTENSIONAHA.112.203661 PMC3684180

[CR44] Wang Z, Moran E, Ding L et al (2014) PPARα regulates mobilization and homing of endothelial progenitor cells through the HIF-1α/SDF-1 pathway. Invest Opthalmol Vis Sci 55:3820. 10.1167/iovs.13-13396 10.1167/iovs.13-13396 PMC4064689 24845641

[CR45] Wenningmann N, Knapp M, Ande A et al (2019) Insights into doxorubicin-induced cardiotoxicity: molecular mechanisms, preventive strategies, and early monitoring. Mol Pharmacol 96:219–232. 10.1124/mol.119.115725 31164387 10.1124/mol.119.115725

[CR46] Yancy CW, Jessup M, Bozkurt B et al (2013) 2013 ACCF/AHA guideline for the management of heart failure: a report of the American College of Cardiology Foundation/American Heart Association Task Force on Practice Guidelines. J Am Coll Cardiol 62:e147–e239. 10.1016/J.JACC.2013.05.019 23747642 10.1016/j.jacc.2013.05.019

[CR47] Yin W-H, Chen J-W, Chen Y-H, Lin S-J (2013) Fenofibrate modulates HO-1 and ameliorates endothelial expression of cell adhesion molecules in systolic heart failure. Acta Cardiol Sin 29:251–260 27122714 PMC4804837

[CR48] Yu CM, Sanderson JE, Shum IOL et al (1996) Diastolic dysfunction and natriuretic peptides in systolic heart failure. Higher ANP and BNP levels are associated with the restrictive filling pattern. Eur Heart J 17:1694–1702. 10.1093/oxfordjournals.eurheartj.a014753 8922918 10.1093/oxfordjournals.eurheartj.a014753

[CR49] Zamorano JL, Lancellotti P, Rodriguez Muñoz D et al (2016) 2016 ESC position paper on cancer treatments and cardiovascular toxicity developed under the auspices of the ESC Committee for Practice Guidelines. Eur Heart J 37:2768–2801. 10.1093/eurheartj/ehw211 27567406 10.1093/eurheartj/ehw211

